# Machine Learning‐Powered Optimization of a CHO Cell Cultivation Process

**DOI:** 10.1002/bit.28943

**Published:** 2025-01-31

**Authors:** Jannik Richter, Qimin Wang, Ferdinand Lange, Phil Thiel, Nina Yilmaz, Dörte Solle, Xiaoying Zhuang, Sascha Beutel

**Affiliations:** ^1^ Institute of Technical Chemistry, Faculty of Natural Sciences Leibniz University Hannover Hannover Germany; ^2^ Institute of Photonics, Faculty of Mathematics and Physics Leibniz University Hannover Hannover Germany

**Keywords:** antibody production, artificial neural network, bioprocess optimization, CHO cells, machine learning

## Abstract

Chinese Hamster Ovary (CHO) cells are the most widely used cell lines to produce recombinant therapeutic proteins such as monoclonal antibodies (mAbs). However, the optimization of the CHO cell culture process is very complex and influenced by various factors. This study investigates the use of machine learning (ML) algorithms to optimize an established industrial CHO cell cultivation process. A ML algorithm in the form of an artificial neural network (ANN) was used and trained on datasets from historical and newly generated CHO cell cultivation runs. The algorithm was then used to find better cultivation conditions and improve cell productivity. The selected artificial intelligence (AI) tool was able to suggest optimized cultivation settings and new condition combinations, which promised both increased cell growth and increased mAb titers. After performing the validation experiments, it was shown that the ML algorithm was able to successfully optimize the cultivation process and significantly improve the antibody production. The best results showed an increase in final mAb titer up to 48%, demonstrating that the use of ML algorithms is a promising approach to optimize the productivity of bioprocesses like CHO cell cultivation processes clearly.

## Graphical abstract

This study investigated the use of a machine learning algorithm to optimize the cultivation of CHO cells used to produce therapeutic monoclonal antibodies. Richter and coworkers developed an artificial neural network that was trained on a very diverse dataset of historical and new cultivation data to find better cultivation conditions. After validation experiments, the final antibody titer was significantly increased, which makes this ML optimization strategy a powerful tool for bioprocess optimizations.

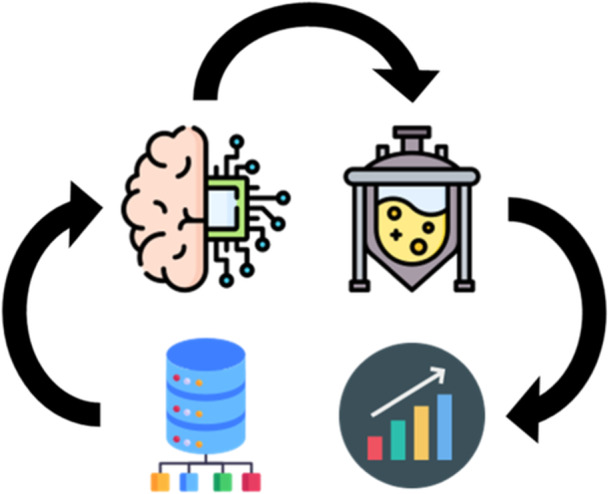

## 1 Introduction

Various artificial intelligence (AI) tools are currently ubiquitous, applied in everyday life, services, industry and research. Also, in biotechnology and bioprocess engineering the possibilities for the development and optimization of processes are thereby enormously expanded. In fact, bioprocesses are highly variable and influenced by a variety of factors such as cell lines, media compositions, process conditions and other environmental influences. This complexity makes manual process optimization and control difficult and would require an enormous amount of experimentation to gain sufficient insight into the various influences on the process (Richter et al. 2023; Scheper et al. 2021). Modern bioreactor systems nowadays allow a high amount of parallel cultivation runs to generate the very diverse and big datasets which are required for AI applications. AI tools are now able to analyze these large amounts of data from bioprocesses, recognize complex patterns as well as predict a wide variety of process courses in silico and determine optimal process conditions. The application of these tools offers the opportunity to effectively manage the upcoming challenges in this field by increasing yields and productivity as well as saving resources, time and money (Kiss, Gottschalk, and Pohlscheidt 2018; Neubauer, Glauche, and Cruz‐Bournazou 2017; Richter et al. 2023; Sinner et al. 2021; Stosch, Portela, and Varsakelis 2021; Udugama et al. 2021).

Machine learning (ML) is one of these AI tools and often synonymously used. ML enables software applications to make more accurate predictions without being explicitly programmed to do so. To predict new output values, machine learning algorithms use historical data as input (Helm et al. 2020; Mahesh 2020; Shinde and Shah 2018). Machine learning is a complex and rapidly evolving field. However, the basic concepts are relatively simple to understand. By understanding the basics of machine learning, it is possible to appreciate the power and potential of this technology. The ability to discover relationships that would be difficult or impossible for humans to find as well as to learn and adapt makes machine learning incredibly versatile, with applications in a wide range of fields. The fact that very large and diverse amounts of data are required and that the exact actions of ML algorithms are complex and difficult for humans to understand (“Black box”) are some disadvantages of this method. Also, the ML algorithm cannot extrapolate beyond the given data ranges, so choosing the right parameters and their values is extremely important. This “data addiction” leads to an important role for the data quality. Furthermore, it is essential to avoid overfitting during the ML training process to not get bad results with new and unknown datasets. (Helm et al. 2020; Mahesh 2020; Shinde and Shah 2018).

Different types of machine learning algorithms are already successfully implemented in the finance sector (Dixon, Halperin, and Bilokon 2020; Nazareth and Ramana Reddy 2023), in manufacturing (Bajic et al. 2018; Razvi et al. 2019; Wuest et al. 2016) or for different retail applications like sales‐forecasting (Krishna et al. 2018) or fruit and vegetable identification (Femling et al. 2018). Furthermore, there are also existing ML algorithms for healthcare applications like cancer prognosis, prediction, and diagnosis (Chugh, Kumar, and Singh 2021; Kourou et al. 2015), drug development (Réda, Kaufmann, and Delahaye‐Duriez 2020) or epilepsy treatment (Abbasi and Goldenholz 2019). In the field of biotechnology and biomanufacturing ML applications are already used in, for example, metabolic engineering (Kim et al. 2020) or analysis of spectroscopic data (Rathore et al. 2023), but also for many other specific topics (Cheng et al. 2023; Duong‐Trung et al. 2023; Helleckes et al. 2023; Mondal et al. 2023; Nikita et al. 2022; Rathore et al. 2023; Walsh et al. 2022).

## 2 Materials and Methods

### 2.1 Cell Line and Media

For this study, a CHO DG44 cell line producing an IgG1 monoclonal antibody (mAb) was used (Sartorius Stedim Cellca GmbH, Göttingen, Germany) and cultured in chemically defined media and feeds of the Sartorius Stedim Cellca platform as described by Schellenberg et al. (2022) and Böhl et al. (2021). The commercially available platform includes a stock medium for the seed culture (SMD) and a production media (PM) for the main culture. Two different feeds were added in for macronutrients such as glucose (feed medium A, FMA) and micronutrients such as amino acids (feed medium B, FMB).

### 2.2 Seed Culture and Small‐Scale Bioreactor Cultivation

The seed cultures were performed as described by Schellenberg et al. (2022) and Böhl et al. (2021). A small‐scale modular bioreactor system (ambr15, Sartorius Stedim Biotech GmbH, Göttingen, Germany) with a working volume of 10–15 mL and online monitoring of dissolved oxygen (DO) concentration and pH was used for the main cultures. All cultivations were performed at a temperature of 36.8°C and stirring speed of 1300 rpm. To prevent foaming, 0.02 µL of a 2% solution of Antifoam C Emulsion (30%, Sigma, Kawasaki, Japan) was automatically added once a day. The main culture process lasted 13 days (Day 0–12). Under standard conditions (STD) this process is inoculated to a cell density of 0.3 × 10^6^ cells/mL, begins then with a 3‐day batch phase and runs with a pH setpoint of pH 7.1–7.2 and a DO setpoint of 60%. The standard feeding scheme consists of a daily feed with FMA and FMB (once a day) from Day 3 on and a special glucose feed to 5 g/L with a 400 g/L glucose solution from Day 5 on, if the measured glucose concentration is below 5 g/L before feeding.

### 2.3 Offline Analytics

Sampling was performed automatically by the Liquid Handler (LH) of the ambr 15 system once per day and then measured manually offline. Concentrations of process‐specific metabolites, including glucose, lactate, glutamine, glutamate, and the product IgG_1_ (mAb) were analyzed using a photometric Cedex Bio Analyzer (Roche, Basel, Switzerland). Analysis of the viable cell density (VCD), the cell viability and the average cell diameter were performed using a trypan blue assay‐based Cedex HiRes Cellcounter and Analyzer system (Roche, Basel, Switzerland).

### 2.4 Calculation of the Cell‐Specific Productivity

The equation for the calculation of the average productivity per cell and day (*Q*_P_) is shown below in Equation (1), similar to Schellenberg et al. (2022) and Meyer et al. (2021):

(1) $$Q_{P}=\frac{\sum_{n=1}^{12}\frac{\left({\text{IgG}}_{n}-{\text{IgG}}_{n-1}\right)}{\left({\text{VCD}}_{n-1}+{\text{VCD}}_{n})/2\times ∆t\right)}}{12}$$

where Δ*t* is the time between two taken and measured samples, here 1 day.

### 2.5 Data Preprocessing and Machine Learning Model Selection

Neural networks have been widely used in various domains, such as image and speech recognition, natural language processing, financial forecasting, and physics simulation (Balas, Roy, and Samui 2017; Gardner and Dorling 1998; Popescu et al. 2009; Wang and Zhuang 2023; Wang et al. 2024). Multilayer Perceptron (MLP) is a feedforward neural network model with layers of interconnected neurons. MLP is capable of recognizing complex data relationships and has been widely used for a variety of tasks, including classification, regression and pattern recognition (Rosenblatt 1958). To investigate the potential relationship between process parameters and performance indicators in CHO cell cultivation, data preprocessing and feature engineering were carried out first, and then model selection was carried out using classical methods such as Linear Regression, Partial Least Squares (PLS), Random Forest (Nonlinear regression) and neural networks (MLP).

**Figure 1:**
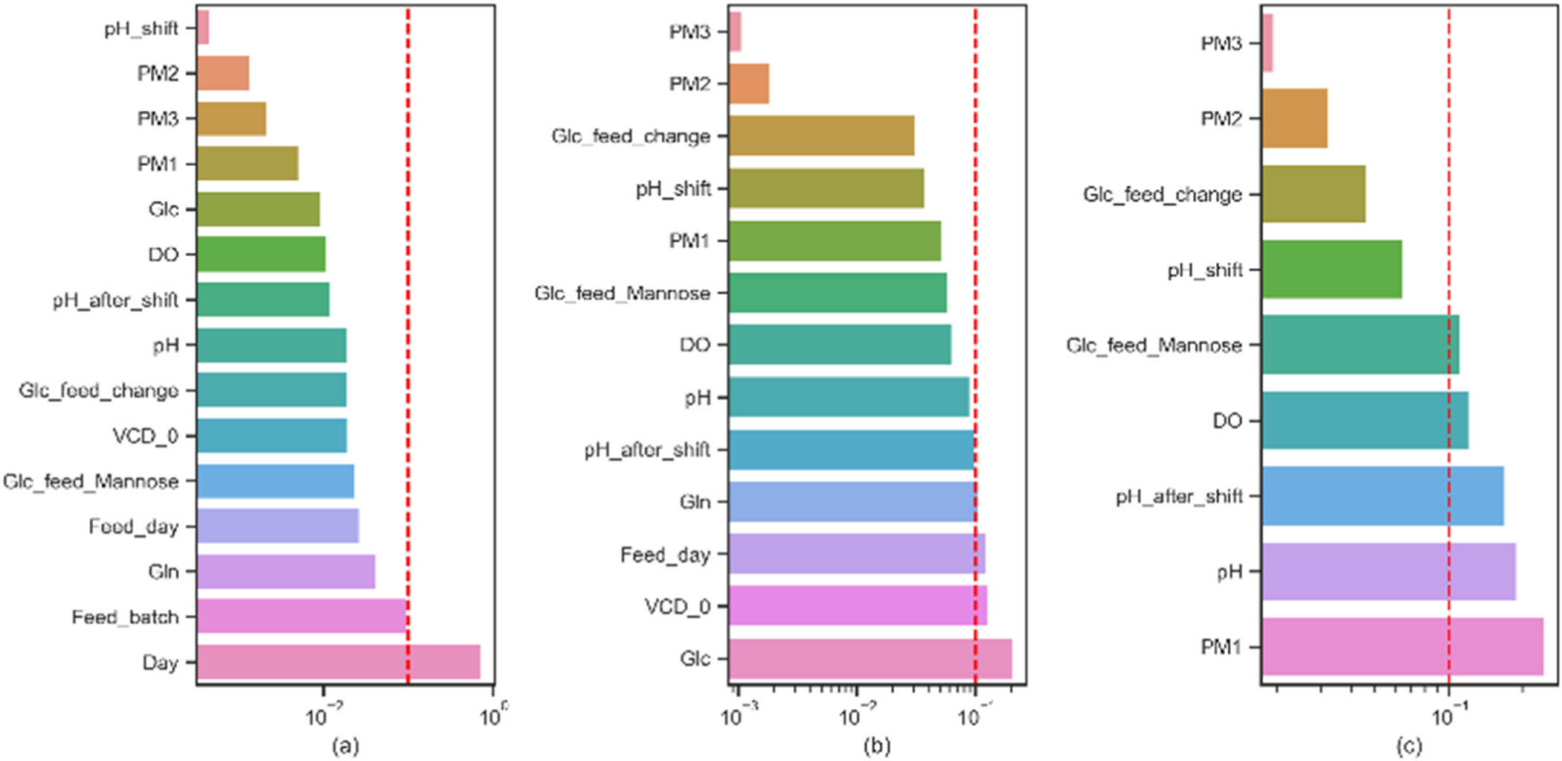
The iterative feature importance analysis to determine the model input with a threshold of 0.1 (red dashed): (a) first iteration; (b) second iteration; (c) third iteration.

Data preprocessing is important to prepare the data for building and training ML models. Here are the preprocessing steps implemented for the bioprocess data:

1. *Data Cleaning*: The irrelevant data or redundant data are removed, and the missing values are handled. In this study, 19 data points were neglected due to contamination or problems with the analytical equipment, resulting in a data set of 735 data points.
2. *Feature Engineering*: Feature importance can reflect how important that feature is for a model in feature engineering and model sensitivity analysis (Guo et al. 2022). The impurity‐based feature importance from the process parameters over the key performance indicator (mAb titer) was computed iteratively after removing the parameters above the threshold (0.1), as shown in Figure 1. After three iterations, the feature importance of the remaining parameters was well below the threshold. The parameters whose feature importance was greater than 0.1 were considered to be the critical process parameters, that is, the input to the machine learning models. Note that “PM1” is neglected due to the imbalanced data distribution.
3. *Train‐Test‐Splitting*: The cleaned data set is splitted into the training (80%) and test (20%) datasets by experience. The training set is used to train a model, while test set is used to evaluate its performance.
4. *Feature Scaling*: The input and output features get normalized to ensure the similar scales for a better model performance. For this study, the training data set was normalized by removing the mean and scaling to unit variance with the StandardScaler from the Scikit‐learn library and the test data set was applied with the identical scalers from the training data set under the i.i.d. assumption (Independent and identically distributed random variables).

In total, 58 cultivation runs including 3 historical STD ones and the 55 runs from Sections 3.1 and 3.2 were selected for the machine learning trial. Each of these 58 cultivation runs consists of 13 data points, that is, limited to one sample taken per day. That means one data point is one time point during one cultivation. This leads to a total data set of 754 data points after data preprocessing. Using feature engineering, key process parameters were selected as model input by feature importance threshold (> 0.1), while the other process parameters were neglected in model training. Finally, each data point contains a series of measurements for both MLP input, that is, DO (setpoint), pH (setpoint), c_Gln_ (start), c_Glc_ (start), pH shift, VCD_0_, feeding start day, glucose feed concentration and process time, and MLP output, that is, VCD_max_, mAb titer, mean cell diameter and mean cell‐specific productivity (*Q*_P_) (see Equation 1). Especially in the case of the cell diameter, it is known that large and old CHO cells are more productive (Lloyd et al. 2000; Pan et al. 2017).

The machine learning model is trained through an optimization process called backpropagation (Linnainmaa 1976), where the model is updated by minimizing the loss function based on the difference between the predicted outputs and the true outputs (Balas, Roy, and Samui 2017; Gardner and Dorling 1998; Popescu et al. 2009).

For regression problems, the most common loss function is the Mean Square Error (MSE), which is written as:

(2) $$L\left(\hat{y},y,W\right)=\frac{1}{2n}\sum_{i=0}^{n}|\hat{y_{i}}-y_{i}{|}_{2}^{2}+\frac{\alpha }{2n}|W{|}_{2}^{2}$$

where $\hat{y_{i}}$ is the predicted value of the i‐th sample, $y_{i}$ is the corresponding true value for total n samples, $\alpha {\left|\left|W\right|\right|}_{2}^{2}$ is an L2‐regularization term that penalizes complex models; and α>0 is a non‐negative hyperparameter that controls the magnitude of the penalty.

In addition to the MSE, *R* ^2^ (coefficient of determination) is also a common regression metric. The best possible value of *R* ^2^ is 1.0 and 0.0 represents the imperfect predictions. It can be negative, because the model can be arbitrarily worse. The estimated *R* ^2^ is defined as:

(3) $$R^{2}\left(y,\hat{y}\right)=1-\frac{\sum_{i=1}^{n}{\left(y_{i}-\hat{y_{i}}\right)}^{2}}{\sum_{i=1}^{n}{\left(y_{i}-\bar{y}\right)}^{2}}$$

where $\bar{y}=\frac{1}{n}\sum_{i=1}^{n}y_{i}$.

The Python language with the Scikit‐learn library was used to build the machine learning models. The Numpy, Pandas and Seaborn libraries were used for the data preprocessing and visualization.

**Figure 2:**
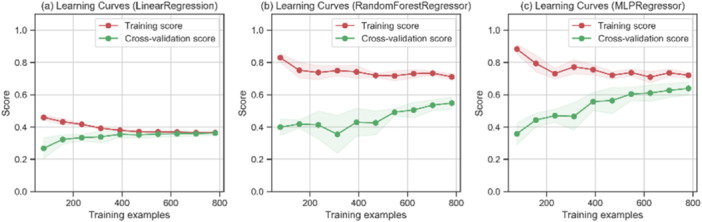
*R* ^2^ score‐based model performance vs*.* training samples between (a) Linear regression (b) Random Forest and (c) MLP.

Figure 2 compares the model performance of Linear Regression, Nonlinear Regression (Random Forest), and Neural Network (MLP) with similar settings based on the *R* ^2^ score. Among the model settings, MLP (MLPRegressor(hidden_layer_sizes = (50, 25, 25, 50))) had the highest *R* ^2^ score (training: 0.721, cross validation: 0.639), which was twice that of Linear Regression (training: 0.366, cross validation: 0.363). As a nonlinear regression model, Random Forest (RandomForestRegressor(n_estimators = 4), training: 0.711, cross validation: 0.548) also performed better than linear regression and PLS (PLSRegression(n_components=4), training: 0.362, cross validation: 0.359). The high nonlinear relationship in the data may explain the poor performance of the linear methods. However, MLP still outperformed Random Forest by 16% in threefold cross validation. Therefore, MLP was selected to model the relationship on the CHO cell culture data set.

To optimize the model performance, the hyperparameters, for example, the number of layers, the number of neurons per layer, the activation function, the backpropagation solver, the learning rate, and so forth, were tuned by iterations. MLPRegressor(hidden_layer_sizes = (100, 50, 25, 50, 100), activation = ‘relu’, solver = ‘adam’, *α* = 0.1) was selected for further investigation after hyperparameters tuning. This MLP model has five hidden layers; For the input layer nine neurons represent the nine critical process parameters selected from the pre‐cultivations (Sections 3.1 and 3.2). For the output layer of the MLP four neurons present the four output key performance indicators. For a data point in the same cultivation run, only process time changes, while the other inputs are fixed within their range of values constrained by the data set.

## 3 Results and Discussion

### 3.1 First Data Collection and Generation

A main step in implementing a machine learning algorithm for a bioprocess is always to explore and collect any historical data that may already be available. Then it is necessary to examine these datasets and determine how diverse the conditions tested were and which parameters were tested to what extent or if only standard conditions were performed. In addition, it is also useful to know if nonoptimal process conditions were used and if the datasets are complete or have gaps. The more useful measurement data available, the better for the subsequent algorithm (Helm et al. 2020; Mahesh 2020).

For this study, several historical datasets of the used CHO cell cultivation process were available, but most of them were performed under standard conditions (STD). For this reason, further cultivation runs were necessary to generate a larger and more diverse data set for creating and training of the appropriate machine learning algorithm. Thereby it was also important to test nonoptimal conditions that lead to worse cultivation performances. This is useful to determine the phase space, which means to cover the widest possible range of variation for the respective parameter and to enable the algorithm for better estimation of the biologically realistic conditions. Furthermore, ML algorithms are not possible to adapt and are bound to their data calibration frame, like all data‐driven methods, so that nonoptimal conditions are also extremely important. The aim of the following experimental setup was also to test a wider value range for some parameters.

**Figure 3:**
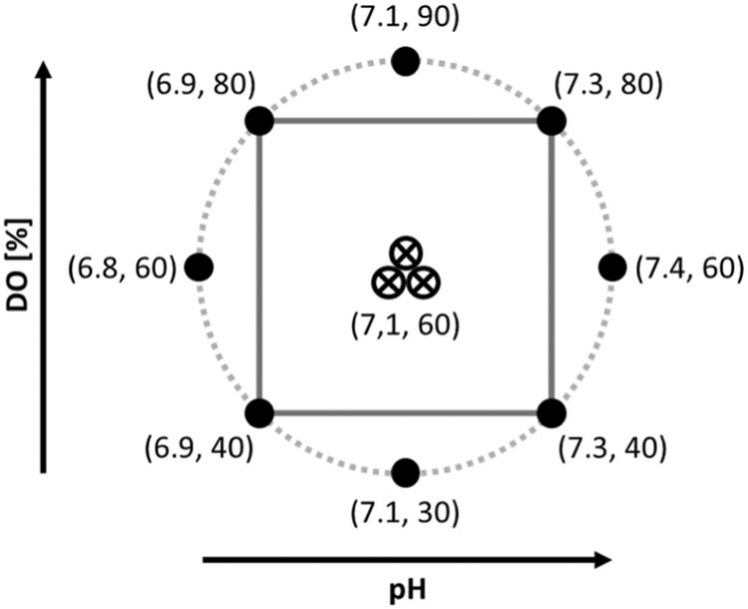
General schematic structure of a Central Composite Circumscribed (CCC) DoE model with two factors and the tested range of the two factors.

So, the first approach was to create an experiment based on a Design of Experiment (DoE). A DoE with two factors and two ranges for each factor was selected for this purpose. The factors to be varied were the pH and the dissolved oxygen concentration (DO). A Central Composite Circumscribed (CCC) structure was selected as the DoE model. This model is a standard approach in the industry, which is particularly suitable for optimizing processes. The setup of the CCC model and the tested parameter ranges are shown in Figure 3.

Furthermore, the pH was also varied in a second way, as the pH was identified as a very sensitive factor in a previous study (Schellenberg et al. 2022). So, cultivation data were also generated in which the pH value was shifted from pH 7.1 to pH 7.3 on four specific days. These experiments were performed in duplicates and the details are shown in Table 1.

**Table 1:** Conditions of the pH shift experiments, tested in duplicates.

Variation	Day	Start pH	End pH
pH shift	0	7.1	7.3
pH shift	3	7.1	7.3
pH shift	4	7.1	7.3
pH shift	5	7.1	7.3

In further vessels the feeding strategy and the medium composition of the cultivation were varied and tested as well. In this case the start concentrations of the two substrates glutamine and glucose in the production media were increased in various stages. In addition, the beginning of the feeding was shifted to earlier or later days in the cultivation time and batch processes completely without feeding were performed. These variations compared to the standard conditions are shown in Table 2.

**Table 2:** Conditions of the experiments with different feeding strategies and medium compositions. Green: higher values compared to the standard conditions, red: lower values compared to the standard conditions.

Variation	c_Glutamine_ (start) [mM]	c_Glucose_ (start) [g/L]	Feeding start day
No feed (Batch)^a^	6	6	‐‐‐
No feed (Batch) & medium	6	7.5	‐‐‐
Higher Gln	9	6	3
Higher Gln	12	6	3
Higher Glc	6	9	3
Higher Glc	6	12	3
Start feeding	6	6	1
Start feeding	6	6	2
Start feeding	6	6	4
Start feeding	6	6	5

^a^ Performed in triplicates.

In total, 34 cultivations were carried out with this very diverse experimental setup. Monoclonal antibody titers and maximal viable cell density (VCD_max_) were the primary focus for evaluating the performance of the various parameter settings and combinations. For this purpose, the final titer at the end of cultivation (Day 12) and the maximum cell density achieved during the entire cultivation process were observed in the vessels. The results of these cultivations and historical data show a first trend for good and bad cultivation conditions. To sum up shortly, the overall best mAb titers were obtained with a higher initial glutamine concentration and earlier feeding. To increase the maximum cell density, it is preferable to start with a higher glucose concentration and to feed the cells earlier. Also, a pH shift on Day 4 led to a higher VCD_max_ compared to STD. Apart from that, the pH shift experiments did not show any major effects on the VCD_max_ or the final mAb titer. Just with a pH shift to pH 7.3 on Day 0 and Day 5, the product titer was even slightly higher than under standard conditions. Poor results in both final mAb titers and maximal VCD were generally obtained with more extreme and low pH values of pH 6.8 and pH 6.9 as well as with late or no feeding (batch). Surprisingly good results in final mAb titer, on the other hand, were also achieved with the setting combinations pH 7.3/40% DO and pH 7.4/60% DO. The detailed results of final mAb titer and VCD_max_ for each of these 34 cultivations can be found in the appendices (Supporting Information S1: Tables 6–8).

### 3.2 Additional Cultivations for Higher Data Diversity

After these initial cultivations, further runs were still necessary to increase the data diversity even more. Firstly, new combinations of the initial four parameters (pH, DO, c_Gln_ (start) and c_Glc_ (start)) were tested, which had more extreme pH and DO values and less glutamine in the start media. So, more stressful and extreme conditions for the CHO cultivation process, especially in terms of pH, DO and c_Gln_ (start), were performed with the expectation of lower VCD_max_ and mAb titer results. These four chosen cultivation settings and their obtained results are shown in Table 3.

**Table 3:** Selected four combinations for additional data with their results for VCD_max_ and mAb titer, in comparison to the standard conditions (STD). Green: higher values compared to the standard conditions, red: lower values compared to the standard conditions. The standard conditions were done in triplicates.

	STD	1	2	3	4
pH	7.1–7.2	7.4	7.3	7.35	7.35
DO [%]	60	90	90	90	90
c_Glc_ (start) [g/L]	6	6.8	6	6	6
c_Gln_ (start) [mM]	6	1	1	1	2
VCD_max_ [Mio. cells/mL]	23.71^a^	21.00	16.86	19.84	22.36
mAb titer [g/L]	3.02^a^	3.56	3.05	3.36	3.55

^a^ Average from triplicates.

As can be seen from the Table 3, the VCD_max_ under standard conditions could not be achieved in any of the four runs. With a range of 16.9–22.4 × 10^6^ cells/mL every run is below the STD result. However, a slight increase in final mAb titers was observed with the tested conditions, which did not necessarily meet the expectations. The titer values of 3.05–3.56 g/L were all higher than the mAb titer obtained with the standard conditions (3.02 g/L). Overall, Experiment 4 showed the best values for the two result parameters. Experiment 2, on the other hand, showed the worst results.

**Figure 4:**
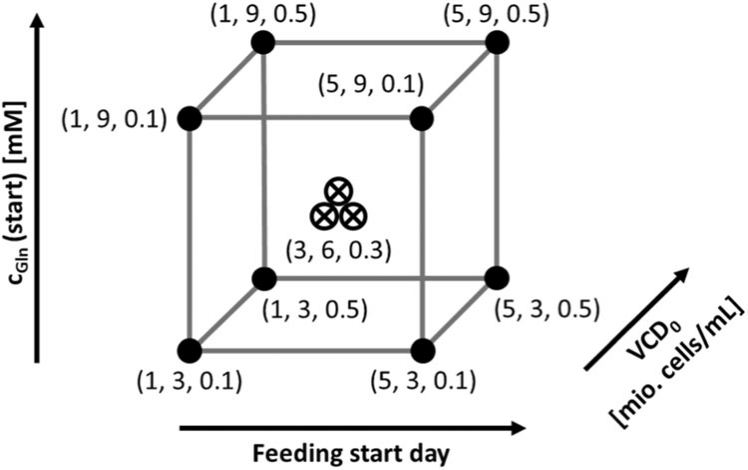
General schematic structure of a full‐factorial DoE model with the tested three factors and two parameter values per factor.

Furthermore, more experiments were necessary to test the additional parameters, which should be incorporated as input parameters into the ML algorithm. Therefore, it was decided to add another four parameters to the previous four parameters, namely: inoculation cell density (VCD_0_), use of pH shifts, feeding start day and glucose feed concentration. For this purpose, a few more cultivation runs with variation of especially VCD_0_ and glucose feeding were necessary. Therefore, it was decided to run another DoE, but this time with three factors (Feeding start day, VCD_0_, and c_Gln_ (start)). Again, a full‐factorial DoE was chosen. The structure is shown in Figure 4, also with the selected value ranges of the three parameters.

In addition to that, further cultivation runs were carried out with variation of the glucose feed. To investigate and determine the influence of this parameter on the process performance, both the condition totally without glucose feed (Glc‐) and with increased glucose feed (Glc + ) were tested. In the second case, glucose was fed to a glucose concentration of 9 g/L before feeding instead of 5 g/L from Day 5 on. These conditions were both tested in triplicates.

By selecting and running these additional experiments, it was possible to identify further trends in process performance because of the various parameters and their values. For example, it could be seen that no experimental setting of the second DoE had a positive effect on the VCD_max_, the best results were just in the same range as the standard conditions. When feeding was started on Day 1 and the VCD_0_ was only 0.1 × 10^6^ cells/mL, the cultures were not as successful in terms of both VCD_max_ and final mAb titer. When feeding was started on Day 5, but inoculation was performed at a cell density of 0.5 × 10^6^ cells/mL, also both parameters were lower compared to the standard conditions. On the other hand, inoculation with a higher cell density of 0.5 × 10^6^ cells/mL and starting the feed from Day 1 had a positive effect on the mAb titer. Final titers of 3.9–4.2 g/L could then be achieved, depending on the starting concentration of glutamine. However, the VCD_max_ in these two experiments was slightly lower than under standard conditions (21–23 × 10^6^ cells/mL). The lack of glucose feeding (Glc−) is also negative for the mAb titer. While the VCD_max_ here is even higher compared to STD, even though the cells die much faster and more severely due to the rapid onset of glucose starvation, these experiments only resulted in a final mAb titer of about 1.8 g/L. Increasing the glucose feed (Glc + ) had only a minimal effect on the mAb titer, which was slightly higher (3.2 g/L) than the standard (3.0 g/L). The detailed results for VCD_max_ and final mAb titer for the second DoE and the Glucose feed experiments can also be found in the appendices (Supporting Information S1: Tables 9 and 10).

In total, these 55 CHO cultivation runs were performed with the ambr15 system, in addition to the historical data, to generate a diverse and big data set for the machine learning trial.

### 3.3 Digital Infrastructure and FAIR‐Database

When processing data, whether manually or with the help of computers or machine learning algorithms, it is essential that the data are consistent, complete and accessible. Applying and complying to the FAIR data principles offers the best opportunity to realize these conditions for the own datasets. FAIR describes a set of guiding principles to make data findable, accessible, interoperable, and reusable. To achieve this, all experiments and their associated data must be provided with metadata, which contain descriptive information about the context, quality, and status or properties of the data. (Boeckhout, Zielhuis, and Bredenoord 2018; Dunning, De Smaele, and Böhmer 2017; European Commission 2018; Stall et al. 2019; Kush et al. 2020; Solle 2020; Tang 2020; Wilkinson et al. 2016; Wise et al. 2019)

During this study, an own FAIR database was created to store and make available the numerous historical and newly generated cultivation datasets in a standardized and complete way, including all meta informations. The JSON data format was chosen for the database, which is based on MongoDB. JSON, which stands for JavaScript Object Notation, is a popular and widely used data format for storing and transmitting structured data. Human‐readability, language‐independence, universality, flexibility and simplicity are the main advantages of the JSON format (Crockford [Youtube] 2011; JSON 2024). Another useful feature is that a JSON schema can be defined in advance, which acts as a template for how the data should look (JSON Schema 2024). Only if the data conform to the specified schema, they are valid and can be uploaded to the database in the next step. This ensures that the data within a data group is standardized uniform. Exemplary excerpts from the JSON database (schema and data) can be seen in the appendices (Supporting Information S1: Figure 7).

The data in the JSON database can then be retrieved and downloaded at any time, or even imported directly into, for example, Python‐based programs or MATLAB and then integrated into simulations, algorithms, or other data processing applications.

### 3.4 ML‐Optimization Trial

After training and testing the ML model (Section 2.5) with the preprocessed data set of the pre‐cultivations, 1 × 10^6^ different combinations of the nine input parameters were generated within the algorithm and for each combination the four output values were predicted. For each input parameter it was possible to have a value within its scaled range. So, for example, one input combination consisted of the eight different cultivation setpoints at one specific cultivation time point (ninth input). After that, this big amount of combinations was sorted by predicted mAb titer values, because the mAb titer was identified as the main output parameter and main target of optimization. If the IgG concentration was predicted to be greater than 4.6 g/L, the corresponding cultivation settings (scaled back input combinations) were saved. A total of 1548 settings, approximately 0.1% of the optimal mAb titer, were used for further validation and study. As expected, all of them were generated with a value of 12 days for the process time input. So, all these 1548 cultivation settings were listed with their predicted final mAb titers and corresponding predicted VCD_max_, maximal average diameter and productivity values. After that, this table was sorted by the best results for each output. Each suggested setting combination was different, so for example the best cultivation settings for the best mAb titer were not automatically the best for maximizing VCD_max_ or *Q*_P_. After sorting the combinations by each of the four output parameters, four cultivation settings were selected from the 10 best results for each output parameter. Care was taken to ensure that these combinations were not too similar to see the influences of the different inputs. To maximize mAb titers, 5 combinations were selected for real validation. These total of 17 selected experiments and their settings are shown in Table 4. Although the conditions for optimizing each output parameter were slightly different, certain tendencies emerged for some input parameters, for example in case of pH, glucose feed and initial glutamine concentration. Within an output block, the conditions for the other five input parameters were often also very similar.

**Table 4:** Selected 17 experiments and their condition combinations to validate the second ML, in comparison to the standard conditions (STD). The process time input is Day 12 for each experiment.

	Name	pH	DO [%]	VCD_0_ [×10^6^ cells/mL]	c_Glc_ (start) [g/L]	c_Gln_ (start) [mM]	Glc feed to	Feed start day	pH shift [7.2 → 7.4]
Top VCD_max_	I a	7.4	40	0.5	6	12	9 g/L	1	on Day 1
	I b	7.4	40	0.5	6	8	9 g/L	1	no
	I c	7.4	40	0.5	6	12	9 g/L	1	no
	I d	7.4	40	0.5	8	12	9 g/L	1	no
Top mAb titer	II a	7.4	40	0.4	6	12	9 g/L	1	no
	II b	7.4	60	0.4	8	12	9 g/L	1	no
	II c	7.4	60	0.4	6	12	9 g/L	1	no
	II d	7.4	40	0.4	10	12	9 g/L	1	no
	II e	7.4	40	0.5	6	12	5 g/L	1	no
Top Avg. diameter	III a	7.4	60	0.4	6	12	9 g/L	1	on Day 2
	III b	7.4	80	0.4	6	12	9 g/L	2	on Day 1
	III c	7.4	60	0.3	6	12	9 g/L	2	on Day 1
	III d	7.4	80	0.3	8	12	9 g/L	4	on Day 1
Top *Q*_P_	IV a	7.4	60	0.2	10	12	9 g/L	4	no
	IV b	7.4	60	0.2	10	12	9 g/L	3	no
	IV c	7.4	40	0.2	10	12	9 g/L	4	no
	IV d	7.4	80	0.3	10	12	9 g/L	4	on Day 1
STD	—	7.2	60	0.3	6	6	5 g/L	3	no

As the ANN is a kind of continuous model modelling possibilities, it can predict the result tendency for the four output parameters and the local optimal predictions may be outside the training range. So, the value ranges of the outputs in the pre‐experiments (Sections 3.1 and 3.2) are not necessary boundaries for the neural network. However, the validation experiments are urgently required to validate the obtained the local optima. These additional experiments are also important to calibrate/update the accuracy of the model out of the range (continuous learning).

### 3.5 Validation Results

**Figure 5:**
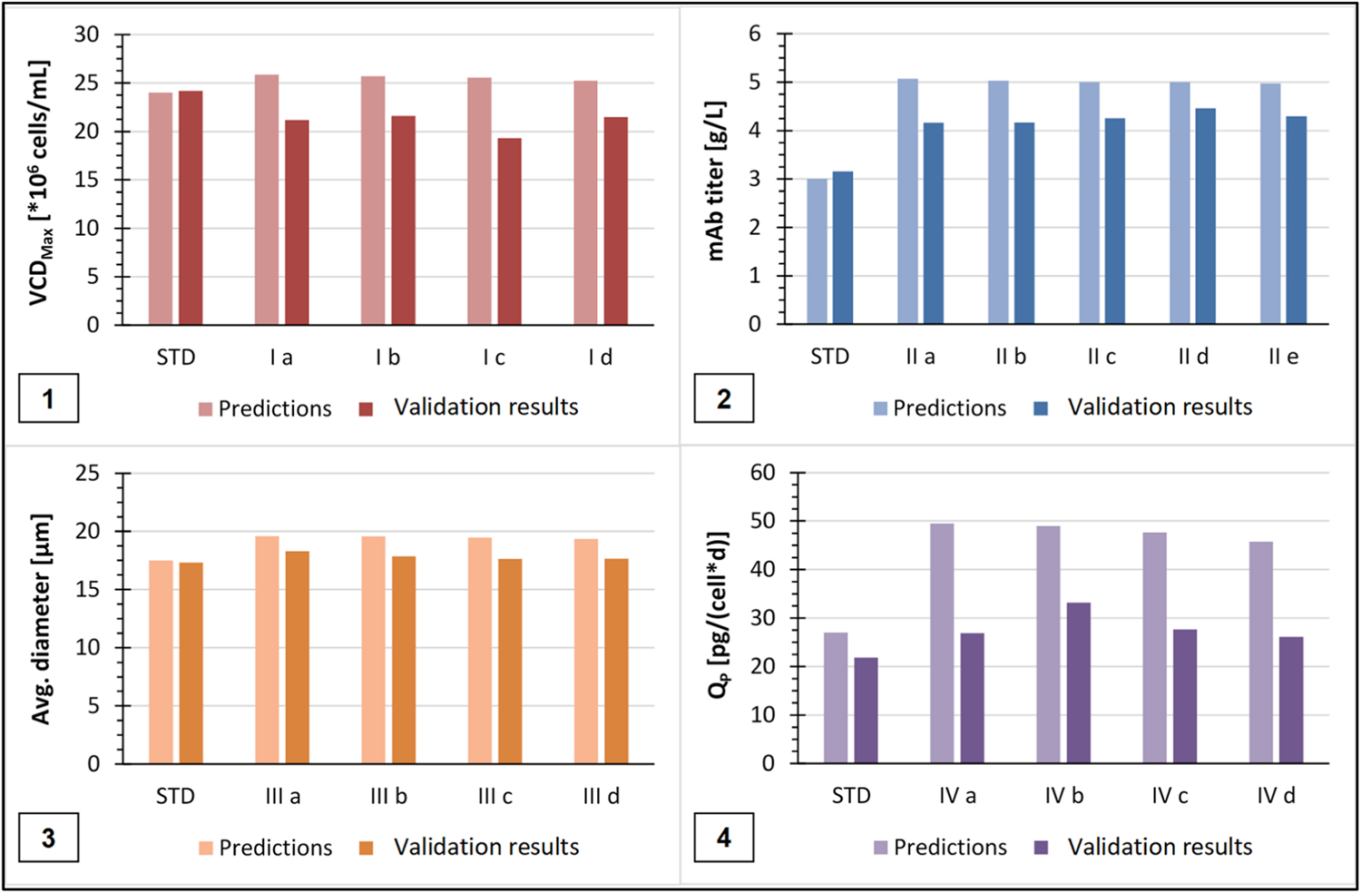
Results of the 17 different validation experiments compared to the ML predictions. In each case also compared to the results and predictions of the standard conditions. (1) Maximal viable cell density (VCD_max_); (2) Final mAb titer; (3) Max. average cell diameter and (4) Average productivity per cell (*Q*_P_).

For the final experiments to validate the ML algorithm and its predictions, these 17 selected setting combinations and three standard runs were performed in the ambr15 system again. The results, comparing the predicted and validated results for the four output parameters, are shown in Figure 5.

**Figure 6:**
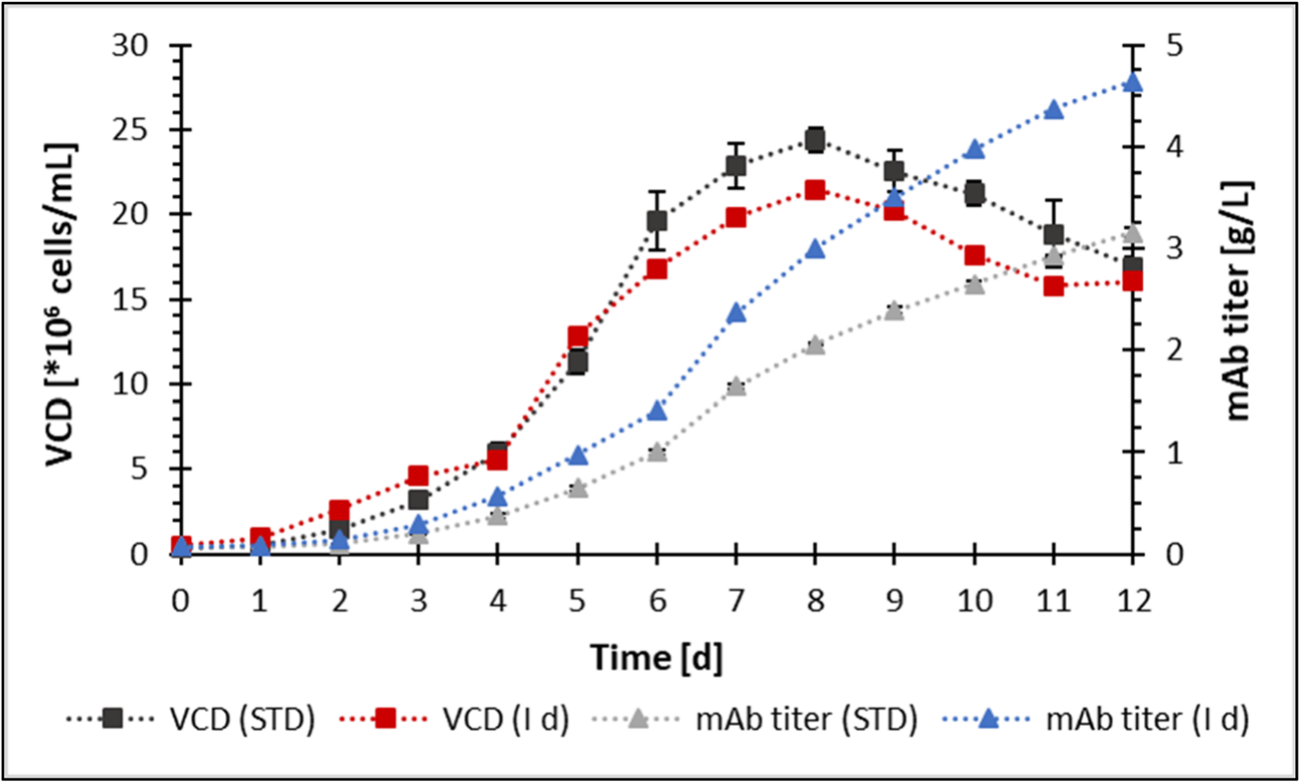
Results of the validation experiments for the viable cell density (VCD) and the mAb titer. Comparison between the cultivations with standard conditions (STD) and with optimal conditions (Experiment I d).

As shown in Figure 5.1, the predicted VCD_max_, which was around 25–26 × 10^6^ cells/mL, was not achieved in any of the four selected experiments. The results obtained were in the range of 19–22 × 10^6^ cells/mL, even lower than the VCD_max_ in STD (24.2 × 10^6^ cells/mL). This can be explained by the fact that the results were initially output according to the maximum mAb titer. This means that possible settings that predict a high VCD_max_ but result in a very low titer were not considered; this was observed for example at Glc− cultivations. Ultimately, however, the highest possible titer should always be achieved. Furthermore, it would not be necessary to run the experiments for 12 days to achieve the highest possible VCD_max_, since the highest cell densities are already reached around Day 8. However, from a process optimization point of view, this does not make much sense as the product concentration is still very low at this point. The absolute best VCD_max_ within the entire series of validation experiments was reached in Experiment II e with 22.2 × 10^6^ cells/mL, which was slightly higher than predicted (21.6 × 10^6^ cells/mL), but still not better than the results from STD. In the case of mAb titer maximization, none of the five selected experiments was able to reach the predicted values of around 5 g/L, but all the real results obtained were clearly higher than the result of the standard runs (3.15 g/L) (Figure 5.2). The best result was obtained in cultivation run II d, where a final mAb titer of 4.46 g/L was achieved. This is a very successful increase of approximately 42%. However, this was not the highest mAb titer achieved in the entire series of validation experiments. Cultivation run I d, which was actually selected to optimize the VCD_max_, achieved a final mAb titer of 4.65 g/L. Compared to the standard run (3.15 g/L), this is an increase of 1.5 g/L or around 48% and therefore a very impressive result. Surprisingly, the predicted mAb titer for this experiment was 4.71 g/L, which was almost achieved, leading also to a higher *Q*_P_ value than predicted. The validation result was therefore within 0.06 g/L (1.3% deviation) of the prediction. Thus, the ML model was able to successfully predict a mAb titer increase which was located outside the training range and could be verified in the validation runs. The conditions and data of this “absolute best” cultivation run regarding to the mAb titer are listed again in Table 5. In addition, the comparison of the cultivation trends in terms of VCD and mAb titer between STD and I d is graphically shown in Figure 6. The increased production of the antibody as well as the lower VCDs from Day 6 on of the optimal conditions run compared to the STD is clearly observable there.

**Table 5:** Suggested cultivation conditions as well as predicted and validated results for the VCD_max_, final mAb titer, maximal cell diameter and cell‐specific productivity of Experiment I d, in comparison to the standard conditions (STD). Green: higher values compared to STD, red: lower values compared to STD. STD were done in triplicates.

**STD**	**Parameters/Inputs**	**Experiment I d**
7.1–7.2	pH	7.4
60	DO [%]	40
0.3	VCD_0_ [× 10^6^ cells/mL]	0.5
6	c_Glucose_ (start) [g/L]	8
6	c_Glutamine_ (start) [mM]	12
5	Glc feed concentration [g/L]	9
12	Cultivation duration [d]	12
no	pH shift	no
3	Feeding start day	1
24.0^a^	Predicted VCD_max_ [× 10^6^ cells/mL]	25.2
24.4^b^	Validated VCD_max_ [× 10^6^ cells/mL]	21.5
3.00^a^	Predicted mAb titer [g/L]	4.71
3.15^b^	Validated mAb titer [g/L]	4.65
17.3^a^	Predicted max. cell diameter [µm]	17.7
17.3^b^	Validated max. cell diameter [µm]	18.1
27.0^a^	Predicted *Q*_P_ [pg/(cell × d)]	25.8
21.9^b^	Validated *Q*_P_ [pg/(cell × d)]	32.2

^a^ The predicted values for STD were not predicted by the ML algorithm. They are based on the knowledge and average values from the historical data and the cultivations which were done in Section 2.

^b^ Average from triplicates.

In addition to these results for VCD_max_ and final mAb titer, Figure 5 also shows the results for ML‐based optimization of maximum average cell diameter (Figure 5.3) and average productivity per cell (*Q*_P_) (Figure 5.4). In the case of cell diameter, it is clearly visible that the predicted maximal diameter of 19.5 µm could not be achieved in any of the four experiments and also not in one of the other experiments of this series. Instead, the real values were in the same range or slightly above the diameter of the standard runs (17.3 µm). The situation is similar for productivity. Again, the predicted productivity of about 46–50 pg/(cell × d) could not be achieved in any of the four selected experiments and also in no other cultivation run here. However, looking at the entire series of experiments an increase to approximately 33 pg/(cell × d) was achieved in cultivation run IV b, compared to 22 pg/(cell × d) in the standard. But the absolute best *Q*_P_ value was achieved in Experiment I c with 34.7 pg/(cell × d). This was higher than predicted for this run (22.9 pg/[cell × d]) and the STD (27.0 pg/[cell × d]), but still not really close to the highest predictions for this value.

The detailed predicted and validated results of final mAb titer, VCD_max_, *Q*_P_ and max. cell diameter for each of the chosen 17 validation experiments can be found in the appendices (Supporting Information S1: Table 11).

These results show that with the current ML algorithm and choice of inputs, it is difficult to both predict and significantly increase the productivity and diameter of the cells. It could be possible that other or additional inputs, not included in this study, are also essential for increasing these two output parameters. Also, the same explanation applies to the optimization of these two outputs as for VCD_max_. The initial filtering by highest mAb titer may not have considered setting combinations that would have predicted a lower titer but even higher productivity or even larger cells, or that would have led to better validation results. Furthermore, it could be generally difficult to use the maximum average cell diameter as an output, because there are multiple factors that affect the diameter of CHO cells (e.g., cell cycle, cell density or chromosome number) (Pan et al. 2019; Pan et al. 2017).

So, to achieve further improvements, especially in terms of more accurate predictions of VCD_max_, productivity or diameter, the algorithm would have to be further adapted/updated and trained on additional, very diverse data. This would require further optimization cycles. It would also be possible to switch from an artificial neural network (ANN) to a convolutional neural network (CNN) by adding more features, which would be deep learning. One possibility for this would be to include microscopic images of the cells. Otherwise, more inputs could be defined (Wohlenberg et al. 2022) or the process could be made more versatile in general, for example, by including other operating modes such as continuous (perfusion mode) or semi‐continuous feeding (Schellenberg 2023). The integration of additional sensors for online or inline monitoring would also be ideal to monitor as many parameters/inputs as possible and to be able to determine their influence on the process performance. This would also raise the amount of data for training of the algorithm.

To investigate whether the conditions optimized in this study and the associated increase in mAb titer can also be achieved in other reactor systems, especially on a larger scale, it would be useful to scale up this process in the next steps to a range of 2–10 L, for example. This would clarify the scale‐up behavior and applicability of these suggested cultivation conditions. In addition, the antibody produced under optimized conditions could subsequently be purified using the standard downstream steps and the bioactivity investigated to determine whether there are any effects caused by the upstream process. However, it has been shown already that changes in the production part of this established mAb production process do not significantly affect the bioactivity of the antibody (Wohlenberg et al. 2022; Wohlenberg et al. 2023).

## 4 Conclusions

The use of a machine learning algorithm proved to be a powerful tool to optimize the given CHO cell culture process. The application was especially successful in increasing the final mAb titer, with an improvement of approximately 48% for the absolute best conditions. This result not only showed a huge increase in product titer but was within 0.06 g/L (1.3% deviation) of the result predicted by the ML algorithm. In the case of VCD_max_ no significant increase was obtained, the real VCD_max_ values were even below the results of the cultivations under standard conditions. The ML predictions could therefore clearly not be achieved here, because only setting combinations with a mAb titer higher than 4.6 g/L were considered. It could be possible that settings for higher predicted and validated VCD_max_ results were not so successful in case of production. The optimization trials of the maximal average cell diameter and the average productivity per cell (*Q*_P_) showed a similar picture. The predictions were not achieved for both inputs. In general, there was only a slight improvement or increase compared to the results under standard conditions.

All in all, these optimization trial only generates better bioprocess conditions for a significant increase in product titer. In case of the other three output parameters, it was not possible to significantly optimize them using the suggested cultivation conditions in the validation experiments. Besides an initial filtering to this output parameters, this could also indicate that further or even completely different input parameters, which have a different or even more direct influence on the respective output, are most likely required to optimize the other three outputs. For this study, only nine input parameters were used, but there are many more parameters that could be investigated and included in the algorithm, especially regarding the metabolism of CHO cells (e.g., adding growth factors or variation/increase of other amino acids).

## Ethics Statement

The authors have nothing to report.

## Consent

The authors have nothing to report.

## Conflicts of Interest

The authors declare no conflicts of interest.

## Supporting information

Supporting information.

## Data Availability

The data that support the findings of this study are available from the corresponding author upon reasonable request.
