# Supplementary material for: Machine Learning‐Powered Optimization of a CHO Cell Cultivation Process
Source: Biotechnol Bioeng. 2025 Jan 31;122(5):1153–64. doi: 10.1002/bit.28943 (PMC11975184; doi:10.1002/bit.28943)
Supplement: Supplementary file 1 — Supporting information. [file BIT-122-1153-s001.docx]

**Appendices**

*Table 6:* Results for VCD_max_ and final mAb titer for the cultivation settings of the first DoE. The other input parameters were not changed compared to STD settings: c_Gln_ (start): 6 mM, c_Glc_ (start): 6 g/L, pH shift: No, VCD_0_: 0.3*10^6^ cells/mL, feed start day: 3, glucose feed concentration: to 5 g/L and process time: 12 days.

| **#** | **pH** | **DO [%]** | **VCD_max_ [*10^6^ cells/mL]** | **Final mAb titer [g/L]** |
| --- | --- | --- | --- | --- |
| 1 | 6.9 | 80 | 17.51 | 2.33 |
| 2 | 6.8 | 60 | 6.57 | 1.06 |
| 3 | 6.9 | 40 | 19.85 | 2.75 |
| 4 | 7.1 | 30 | 25.52 | 3.37 |
| 5 | 7.1 | 90 | 23.72 | 3.07 |
| 6 | 7.3 | 40 | 22.42 | 3.71 |
| 7 | 7.3 | 80 | 21.34 | 3.43 |
| 8 | 7.4 | 60 | 23.24 | 3.76 |
| 9^†^ | 7.1 | 60 | 24.84 | 3.10 |
| 10^†^ | 7.1 | 60 | 25.58 | 3.32 |
| 11^†^ | 7.1 | 60 | 25.27 | 3.76 |

† STD runs.

*Table 7:* Results for VCD_max_ and final mAb titer for the cultivation settings of the pH shift experiments. The STD results are the same as from the first DoE (Table 6). The other input parameters were not changed compared to STD settings: DO (setpoint): 60%, c_Gln_ (start): 6 mM, c_Glc_ (start): 6 g/L, VCD_0_: 0.3*10^6^ cells/mL, feed start day: 3, glucose feed concentration: to 5 g/L and process time: 12 days.

| **#** | **Start pH** | **End pH** | **Day** | **VCD_max_  [*10^6^ cells/mL]** | **Final mAb titer [g/L]** |
| --- | --- | --- | --- | --- | --- |
| 12 | 7.1 | 7.3 | 0 | 23.81 | 3.49 |
| 13 | 7.1 | 7.3 | 0 | 22.53 | 3.41 |
| 14 | 7.1 | 7.3 | 3 | 23.88 | 3.20 |
| 15 | 7.1 | 7.3 | 3 | 24.57 | 3.49 |
| 16 | 7.1 | 7.3 | 4 | 23.07 | 3.44 |
| 17 | 7.1 | 7.3 | 4 | 28.54 | 3.16 |
| 18 | 7.1 | 7.3 | 5 | 22.64 | 3.50 |
| 19 | 7.1 | 7.3 | 5 | 21.35 | 3.43 |

*Table 8:* Results for VCD_max_ and final mAb titer for the cultivation settings of the media variation experiments. The other input parameters were not changed compared to STD settings: DO (setpoint). 60%, pH (setpoint): pH 7.2, pH shift: No, VCD_0_: 0.3*10^6^ cells/mL, glucose feed concentration: to 5 g/L and process time: 12 days.

| **#** | **c_Gln_ (start) [mM]** | **c_Glc_ (start)**  **[g/L]** | **Feed start day** | **VCD_max_  [*10^6^ cells/mL]** | **Final mAb titer [g/L]** |
| --- | --- | --- | --- | --- | --- |
| 20 | 6 | 6 | --- | 6.89 | 0.45 |
| 21 | 6 | 6 | --- | 7.39 | 0.42 |
| 22 | 6 | 6 | --- | 7.57 | 0.44 |
| 23 | 6 | 7.5 | --- | 6.93 | 0.46 |
| 24 | 9 | 6 | 3 | 20.98 | 4.14 |
| 25 | 12 | 6 | 3 | 21.94 | 3.88 |
| 26 | 6 | 9 | 3 | 22.00 | 3.31 |
| 27 | 6 | 12 | 3 | 21.19 | 3.74 |
| 28 | 6 | 6 | 1 | 19.18 | 4.17 |
| 29 | 6 | 6 | 2 | 22.46 | 3.97 |
| 30 | 6 | 6 | 4 | 20.99 | 3.00 |
| 31 | 6 | 6 | 5 | 11.01 | 0.87 |
| 32^†^ | 6 | 6 | 3 | 22.04 | 3.16 |
| 33^†^ | 6 | 6 | 3 | 21.60 | 3.36 |
| 34^†^ | 6 | 6 | 3 | 20.40 | 2.79 |

† STD runs.

*Table 9:* Results for VCD_max_ and final mAb titer for the cultivation settings of the second DoE. The STD results here are already shown as averages in Table 3 in the main document. The other input parameters were not changed compared to STD settings: DO (setpoint). 60%, pH (setpoint): pH 7.2, c_Glc_ (start): 6 g/L, pH shift: No, glucose feed concentration: to 5 g/L and process time: 12 days.

| **#** | **c_Gln_ (start) [mM]** | **VCD_0_  [*10^6^ cells/mL]** | **Feed start day** | **VCD_max_  [*10^6^ cells/mL]** | **Final mAb titer [g/L]** |
| --- | --- | --- | --- | --- | --- |
| 35 | 9 | 0.1 | 1 | 12.46 | 2.44 |
| 36 | 9 | 0.5 | 1 | 23.09 | 4.24 |
| 37 | 9 | 0.1 | 1 | 23.71 | 3.15 |
| 38 | 9 | 0.5 | 1 | 21.66 | 2.82 |
| 39 | 3 | 0.1 | 5 | 8.77 | 1.74 |
| 40 | 3 | 0.5 | 5 | 21.07 | 3.89 |
| 41 | 3 | 0.1 | 5 | 23.52 | 2.78 |
| 42 | 3 | 0.5 | 5 | 19.49 | 2.58 |
| 43^†^ | 6 | 0.3 | 3 | 21.24 | 2.93 |
| 44^†^ | 6 | 0.3 | 3 | 24.87 | 3.02 |
| 45^†^ | 6 | 0.3 | 3 | 25.03 | 3.11 |

† STD runs.

*Table 10:* Results for VCD_max_ and final mAb titer for the cultivation settings of the glucose feed variation experiments (Glc+ and Glc-). The STD results are same as from the second DoE (Table 9). The other input parameters were not changed compared to STD settings: DO (setpoint). 60%, pH (setpoint): pH 7.2, c_Gln_ (start): 6 mM, c_Glc_ (start): 6 g/L, pH shift: No, feed start day: 3 and process time: 12 days.

| **#** | **Glucose feed** | **VCD_max_ [*10^6^ cells/mL]** | **Final mAb titer [g/L]** |
| --- | --- | --- | --- |
| 46 | No (Glc-) | 24.74 | 1.82 |
| 47 | No (Glc-) | 23.77 | 1.79 |
| 48 | No (Glc-) | 29.40 | 1.84 |
| 49 | to 9 g/L (Glc+) | 21.62 | 3.18 |
| 50 | to 9 g/L (Glc+) | 24.52 | 3.37 |
| 51 | to 9 g/L (Glc+) | 24.66 | 3.18 |


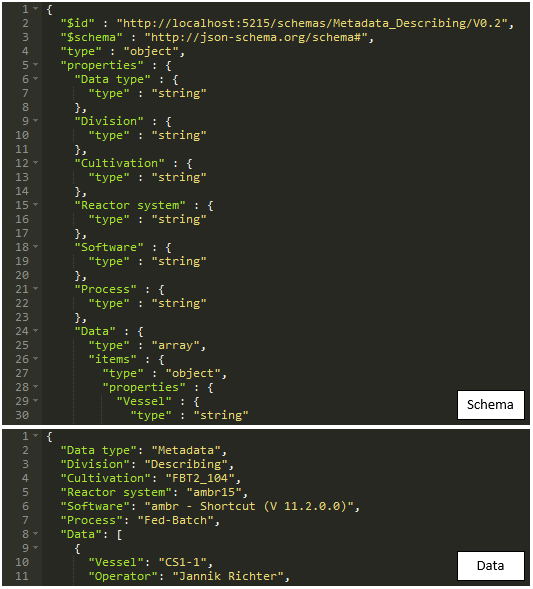


*Figure 7:* Exemplary excerpts from the JSON database. The JSON schema (above) and the JSON data of a metadata data set which fulfills the schema (below).

*Table 11:* Predicted and validated results of the 17 chosen experiments for the ML algorithm validation.

| **#** | **VCD_max_**  **[*10^6^ cells/mL]** | | **Final mAb titer [g/L]** | | **Q_P_**  **[pg/(cell*d)]** | | **Max. cell diameter [µm]** | |
| --- | --- | --- | --- | --- | --- | --- | --- | --- |
|  | ***Predict.*** | ***Validat.*** | ***Predict.*** | ***Validat.*** | ***Predict.*** | ***Validat.*** | ***Predict.*** | ***Validat.*** |
| ***I a*** | 25.84 | 21.18 | 4.67 | 4.24 | 27.87 | 27.32 | 18.69 | 17.84 |
| ***I b*** | 25.72 | 21.59 | 4.83 | 4.40 | 26.55 | 31.53 | 18.00 | 18.25 |
| ***I c*** | 25.56 | 19.31 | 4.96 | 4.50 | 22.90 | 34.73 | 18.15 | 18.07 |
| ***I d*** | 25.24 | 21.49 | 4.71 | 4.65 | 25.78 | 32.19 | 17.68 | 18.07 |
| ***II a*** | 25.18 | 21.83 | 5.07 | 4.16 | 23.39 | 27.80 | 18.67 | 18.07 |
| ***II b*** | 23.45 | 18.86 | 5.03 | 4.17 | 19.38 | 28.75 | 18.35 | 18.04 |
| ***II c*** | 23.64 | 19.35 | 5.00 | 4.26 | 18.95 | 26.98 | 18.90 | 18.02 |
| ***II d*** | 23.23 | 18.74 | 5.00 | 4.46 | 26.72 | 34.65 | 17.61 | 17.98 |
| ***II e*** | 21.57 | 22.20 | 4.97 | 4.30 | 19.10 | 30.60 | 17.99 | 18.25 |
| ***III a*** | 24.25 | 18.15 | 4.62 | 4.10 | 25.71 | 32.99 | 19.60 | 18.28 |
| ***III b*** | 24.87 | 19.56 | 4.60 | 4.14 | 20.08 | 31.17 | 19.57 | 17.84 |
| ***III c*** | 23.35 | 20.78 | 4.62 | 4.19 | 29.16 | 27.31 | 19.48 | 17.62 |
| ***III d*** | 22.89 | 18.53 | 4.61 | 3.67 | 38.76 | 27.87 | 19.34 | 17.65 |
| ***IV a*** | 23.46 | 19.77 | 4.61 | 3.69 | 49.52 | 26.84 | 18.32 | 17.46 |
| ***IV b*** | 22.14 | 19.69 | 4.63 | 4.12 | 48.96 | 33.17 | 18.25 | 17.63 |
| ***IV c*** | 22.98 | 19.38 | 4.67 | 3.79 | 47.70 | 27.63 | 17.81 | 17.49 |
| ***IV d*** | 23.44 | 18.09 | 4.60 | 3.48 | 45.80 | 26.12 | 18.79 | 17.65 |
